# Dr. William M. Boling

**Published:** 1859-07

**Authors:** 


### NECROLOGICAL NOTICE.

DR. WILLIAM M. BOLING, of Montgomery, Alabama.—We announced in our last number the death of Dr. Boling, which took place on the fourth of March, 1859, after a lingering illness. Since then we have learned from our esteemed collaborator, Dr. Bozeman, who knew him intimately for many years, that Dr. Boling was born in Baltimore County, Maryland, on the fourteenth of July, 1811, and when eighteen years of age moved to Greensburg, Pennsylvania, having at the time nothing more than an ordinary English education. While here he made choice of the profession of Medicine, and in 1833 entered upon its study in the office of Dr. S. P. Brown.

He prosecuted his studies with great assiduity, applying himself not only to his text books, but to the Greek, Latin, and French languages, a very good knowledge of all of which he soon acquired. He attended his first course of lectures in the Jefferson Medical College, 1835-6, and soon after the close of the session he commenced to practice in Coosawda, Antauga County, Alabama, where he remained until the fall of 1837, when he married, and returned to Philadelphia, to attend his second course of lectures in the Jefferson College. After graduating the following spring he returned to Alabama, and settled in Montgomery, where he continued to reside up to the time of his death.

As to the success Dr. Boling met with in his profession, and the position he occupied as a writer and teacher, we have only to refer to the periodicals of the day. His contributions to the medical literature of our country were always well received. Many of his papers possess great merit, and none, perhaps, more than the one entitled "Observations on Remittent Fever," published in the *American Journal of the Medical Sciences*. In this essay he has shown most clearly the wide range of his thought, the shrewdness of his intellect, the force of his reasoning powers, and the knowledge he possessed not only of the resource of his art, but of nature herself. His views here set forth have been largely quoted by nearly, if not all, subsequent writers upon the subject in this country.

In 1848 he was invited to the Chair of Materia Medica in the Memphis Medical School, which he accepted, but filled only one session. He gave, however, ample proof of his ability as a teacher, and endeared himself to a wide circle of friends throughout the South and West.

Soon after leaving Memphis he received a call from the Transylvania Medical School, to the Chair of Obstetrics. In this school he delivered one course of lectures, (1849–50,) and as a thorough teacher of the branch assigned him he gave entire satisfaction. After the session closed he resigned his chair and returned to his home in Alabama.

There are other positions which he was called upon to fill, highly complimentary; such as President of the Alabama State Medical Association, and Vice-President of the American Medical Association. He never accepted a professorship in any other school after he left Transylvania, although several advantageous offers were made him. Having a wide circle of friends, he preferred remaining among them to being a public teacher. The result of this was an overwhelming practice. For several years he bore up under his immense physical and mental labor, but at last his feeble frame began to give way under it. In the spring of 1853 paraplegia showed itself, from which, although better at times, he never recovered. Notwithstanding the gravity of this infirmity he continued, as long as he was able, to do some practice, and never ceased to take that interest in medicine which had characterized the whole course of his professional life. His disease gradually grew upon him until last winter, when he became almost completely helpless, and finally died from the prostrating effect of nausea and vomiting occasioned by an attack of intermittent fever.

Thus ended the short but useful career of a man, who may truly be said to have been a martyr to his noble calling. Had he enjoyed the advantages of many, and been more fortunate in the selection of a wider field for his labors, we can safely assert he would have been second to no one in the ranks of his profession.

The private character of Dr. Boling was spotless. His moral worth, coupled with the kind and generous impulses of his heart, rendered him one of the noblest of men. His intercourse with the world was always characterized by the strictest integrity. He loved truth and candor. To many his manner appeared austere, though not to such an extent as to render him unapproachable. Dignified and self-possessed, he always commanded respect. In the relation of husband and father, the goodness of his nature was ever dominant; gentle and affectionate, he smoothed the asperities of life and diffused happiness around him. His name will long be cherished by his many friends.

